# Developmental and architectural principles of the lateral-line neural map

**DOI:** 10.3389/fncir.2013.00047

**Published:** 2013-03-26

**Authors:** Jesús Pujol-Martí, Hernán López-Schier

**Affiliations:** Research Unit of Sensory Biology and Organogenesis, Helmholtz Zentrum MünchenNeuherberg, Munich, Germany

**Keywords:** lateral line, Mauthner, behavior, neural circuit, hair cells, auditory, somatotopy

## Abstract

The transmission and central representation of sensory cues through the accurate construction of neural maps is essential for animals to react to environmental stimuli. Structural diversity of sensorineural maps along a continuum between discrete- and continuous-map architectures can influence behavior. The mechanosensory lateral line of fishes and amphibians, for example, detects complex hydrodynamics occurring around the animal body. It triggers innate fast escape reactions but also modulates complex navigation behaviors that require constant knowledge about the environment. The aim of this article is to summarize recent work in the zebrafish that has shed light on the development and structure of the lateralis neural map, which is helping to understand how individual sensory modalities generate appropriate behavioral responses to the sensory context.

## INTRODUCTION

The sensation of external stimuli is essential for all life forms to react appropriately to environmental cues. Even the simplest animals are endowed with sensory systems. For example, changes in light intensity directly affect the activity of cilia in the photoreceptor cells of sponge larvae, which influences local motor responses (Leys and Degnan, 2001). It is the higher probability of a biased individual action of ciliary movement that directs swimming along the luminosity gradient. In more complex animals, the evolution of neurons and a centralized nervous system allows sensory organs to control the activity of motor centers in a coordinated manner, thereby improving the speed and accuracy of sensory-motor transformations (Moroz, 2009; Jékély, 2011; Arber, 2012; Northcutt, 2012). The meaningful use of dynamic sensory stimuli is a complex computational problem. Sensory systems can optimize a solution by combining the acquisition and processing of the quality, quantity and spatial distribution of the stimuli (Chaudhari and Roper, 2010; DeMaria and Ngai, 2010; Lumpkin et al., 2010; Schwander et al., 2010; Sung and Chuang, 2010; Chacron et al., 2011). This can be achieved via two architectural properties of the sensory systems: the structure of the peripheral receptors, which is important for information acquisition and filtering, and the neural representation of the information, which is essential for consistent sensory processing.

High-order processing of sensory information largely relies on the accurate construction of spatially arranged neuronal projections, known as neural maps (Gardner and Martin, 2000; Luo and Flanagan, 2007; Feldheim and O’Leary, 2010; Imai et al., 2010). Neural maps appear to be a universal solution to this problem because they are present in phylogenetically distant animals and in diverse sensory systems (Knudsen et al., 1987; Kaas, 1997; Weinberg, 1997). For example, the visual system of invertebrates and vertebrates exhibits a neural projection structure known as retinotopic map, in which nearby positions in the sensory region project afferent neurons onto nearby regions of the brain (Lemke and Reber, 2005; Feldheim and O’Leary, 2010). Neural maps showing this arrangement encode positional information and are called continuous or topographic. Other sensory systems show qualitatively different map architecture, known as discrete, whereby discrete information such as stimulus identity is separately represented in the brain (Luo and Flanagan, 2007). This is the case of the olfactory systems in which each odor is encoded by a unique ensemble of neurons without any spatial arrangement in relation to the olfactory area (Imai et al., 2010). Some sensory modalities, however, construct neural maps that fall between the discrete and the continuous (topographic; Scheich, 1991).

The superficial mechanosensory lateral line in fishes and amphibians combines some structural and physiological characteristics of the mammalian vestibulo-auditory and somatosensory systems. It comprises the three basic elements of a vertebrate sensory system: peripheral receptors, intermediate afferent transmission elements, and central processing units (Winklbauer, 1989; Ghysen and Dambly-Chaudière, 2007; Bleckmann and Zelick, 2009). The peripheral organs are a group of mechanoreceptive neuromasts, which locally acquire mechanical signals using their sensory elements called hair cells. The spatial sensitivity of the lateral line is about one body length from the surface of the animal, for which it also receives the name of “sense of distant touch” (Engelmann et al., 2000; Coombs, 2001). Hair cells transform mechanical stimuli into chemical signals that are further converted into electrical impulses that lateralis afferent neurons transport to the brain (Hudspeth, 1989; Chagnaud et al., 2007; Bleckmann and Zelick, 2009; Schwander et al., 2010). This first-order neuronal population projects central axons to contact second-order output neurons located in the medial octavolateralis nucleus (MON) of the hindbrain. From this first relay center, most lateral-line information is transmitted to the torus semicircularis (TS) in the midbrain (Bleckmann, 2008). The TS is equivalent to the mammalian inferior colliculus, which is a major target of auditory information (Barrett, 1973; Wubbels and Schellart, 1997).

The lateral line is able to localize fast-changing mechanical signals in three dimensions around the animal’s body and provides essential sensory information for centrally controlled complex motor behaviors such as navigation, schooling, rheotaxis, and prey tracking (Montgomery et al., 1997,^2000^; Coombs et al., 1998; Bleckmann, 2008; Goulet et al., 2008; Bleckmann and Zelick, 2009; Chaudhari and Roper, 2010). It also mediates fast reflex reactions mediated by a large reticulospinal neuron called Mauthner cell (Zottoli and Van Horne, 1983; Metcalfe et al., 1985). The lateral line, therefore, is a prime example of a sensory system that commands contrasting behaviors in response to a unique sensory cue: mechanical fluctuations of the surrounding fluid (Engelmann et al., 2000; Chagnaud et al., 2007). How does the architecture of the lateralis neural map allow for a robust and accurate information flow from the sensory receptors to brain areas underlying the different behaviors? In this review we will discuss this question using recent studies in the zebrafish that provide important insights about lateral-line neural map development and architecture. We will also use the current knowledge and technological state-of-the-art to anticipate research directions of this important problem in neurobiology.

## BACKGROUND

### DEVELOPMENT AND ORGANIZATION OF THE LATERAL-LINE SENSORY RECEPTORS

**FIGURE 1:**
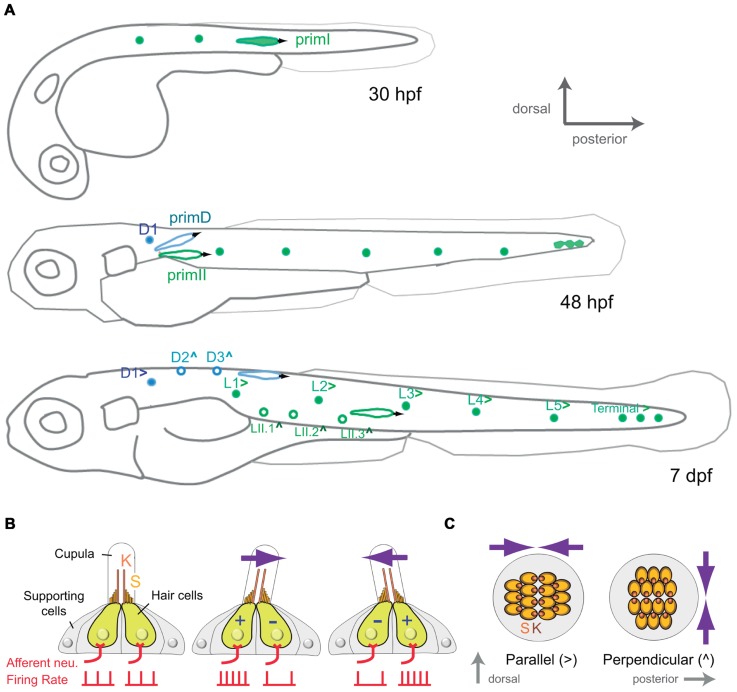
**Development and organization of the posterior lateral-line sensory receptors**. **(A)** Lateral view of a developing zebrafish at around 30, 48 hpf and 7 dpf (days post-fertilization) showing the development of the mechanoreceptive neuromasts that form the posterior lateral line. primI, first primordium; primII, second primordium; primD, dorsal primordium. (>) and (^∧^) indicate parallel and perpendicular neuromasts, respectively. **(B)** Lateral view of a neuromast. The hair-bundle comprises the kinocilium (K) and the stereocilia (S), and is contained within a gelatinous cupula. A neuromast contains two populations of hair cells of opposing hair-bundle polarities. Thus, a water movement (blue arrow) bending the cupula in a given direction depolarizes (+) one population of hair cells, whereas hyperpolarizes (-) the other one. This is translated into an increase or a decrease of the firing rate of the afferent neuron associated to each hair-cell population. **(C)** Top view of a parallel and a perpendicular neuromast, which are sensitive to water movements across orthogonal axes.

Most of what we know about the development and organization of the zebrafish lateral line comes from studies on the posterior aspect of the system, which comprises all the elements associated to the neuromasts on the trunk of the animal. The posterior lateral line develops early during embryogenesis from bilateral cephalic placodes. At around 18 hpf (hours post-fertilization) a first placode appears just caudal to the ear and gives rise to neuroblast precursors of the lateralis afferent neurons, and to a moving group of cells known as first primordium (primI; **Figure 1A**). At around 20 hpf the primI begins to migrate toward the tail along the horizontal myoseptum. During a journey lasting about 20 h it deposits an average of eight cellular rosettes that will eventually differentiate into neuromasts (Ghysen and Dambly-Chaudière, 2007). Few hours after primI, a second placode arises at the same place of origin of primI. This placode splits into several groups of cells. One group directly differentiates on location as the so-called D1 neuromast, which together with primI-derived neuromasts form the primary posterior lateral line. The two other groups of cells form the migratory second and dorsal primordia (primII and primD). PrimII deposits two or three neuromasts along the trail of primI. PrimD follows an upward path to produce two neuromasts on the dorsum of the fry. These later-born neuromasts form the secondary posterior lateral line (**Figure 1A**; Ghysen and Dambly-Chaudière, 2007).

A mature neuromast consists of a core of 20–30 mechanosensory hair cells surrounded by a similar number of non-sensory supporting cells (**Figure 1B**). Hair cells derive their name from the hair bundle, a mechanosensing organelle that protrudes from the cell’s apical surface. In the neuromast, hair bundles are contained within a gelatinous cupula that projects into the surrounding water. The hair bundle is formed by an array of stereocilia arranged in rows of increasing length, like a staircase, and a kinocilium eccentrically located adjacent to the tallest stereocilia. In this way, each hair bundle and thus, each hair cell, is polarized within the plane of the neuromast epithelium (Duvall et al., 1966; Ghysen and Dambly-Chaudière, 2007). A mechanical deflection of the stereocilia toward the kinocilium depolarizes the cell, whereas a deflection away from the kinocilium hyperpolarizes it (Hudspeth, 1989). Therefore, the polarity of hair bundle endows hair cells with vectorial excitability. Each neuromast contains two intermingled populations of hair cells, equal in number, whose stereocilia are oriented along a single axis but in opposite directions (**Figures 1B** and **C**; Rouse and Pickles, 1991; López-Schier et al., 2004). Thus, each neuromast is mechanically bidirectional sensitive. Furthermore, two types of neuromasts have been described according to the planar polarization of their hair cells. Parallel neuromasts are oriented along the animal’s anteroposterior body axis, whereas perpendicular neuromasts orient orthogonally (**Figure 1C**; López-Schier et al., 2004). Parallel and perpendicular neuromasts originate from different primordia. While all primI-derived neuromasts and the D1 neuromast are parallel, primII-derived neuromasts and those deposited by primD are perpendicular. Consequently, the posterior lateral line from the one-week old zebrafish larva consists of about 14 neuromasts that occupy stereotypical positions covering the dorsal and lateral aspects of the fish’s trunk and that are able to locally detect bidirectional mechanical signals at orthogonal axes (**Figure 1A**).

### DEVELOPMENT OF THE LATERALIS AFFERENT NEURONS

**FIGURE 2:**
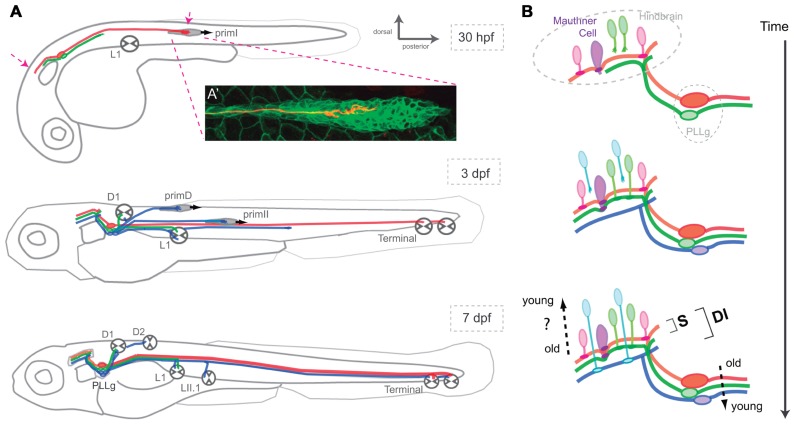
**Assembly of the posterior lateral-line neural map**. **(A)** Lateral view of a developing zebrafish at around 30 hpf, 3 and 7 dpf showing the coincident progressive development of the lateralis afferents and their peripheral targets. Arrows at 30 hpf indicate growing lateralis afferent’s central and peripheral axons. PLLg: posterior lateralis ganglion formed by the lateralis afferent’s cell bodies. **(A’)** A single lateralis afferent neuron is labeled in red in a transgenic zebrafish embryo expressing GFP in primI. The peripheral axonal growth cone can be observed within the migrating primordium. **(B)** Lateral view of the developing hindbrain and lateralis afferent’s central axons at around 30 hpf, 3 and 7 dpf depicting an hypothetical temporal code that matches lateralis afferents with second-order neurons that are born at similar times. Both in **(A)** and **(B)**, red and green lateralis afferents belong to the first neuronal subclass which projects dorsal axons that contact the Mauthner cell. Red and green neurons innervate posterior and anterior primary neuromasts, respectively. Blue lateralis afferents belong to the second neuronal subclass which projects ventrolateral axons that do not contact the Mauthner cell. The two neuronal subclasses form a dimorphic neural map (DI) whereas only the neurons of the first subclass shape the somatotopic map (S). Neurons in red are the first-born neurons whereas neurons in blue are the latest-born neurons. primI, first primordium; primD, dorsal primordium; primII, second primordium. L1, Terminal and D1 neuromasts are primary and parallel (>). LII.1 and D2 neuromasts are secondary and perpendicular (^∧^).

Pioneering studies on the development of the lateral line in the zebrafish have shown that the first placode, which gives rise to primI, also generates lateralis afferent neurons. Lateralis afferents begin to project central and peripheral axons concurrently as soon as they differentiate. Growing central axons extend toward the hindbrain whereas peripheral axons project growth cones that follow the migrating primI and eventually innervate each deposited neuromasts (**Figure 2A**; Gompel et al., 2001; Ghysen and Dambly-Chaudière, 2007). The use of newly developed tools for cellular birth dating has shown that lateralis neurogenesis during embryonic development occurs in two discrete waves (Sarrazín et al., 2010). The earliest differentiating neurons extend their peripheral axons to the adjacent primI, which expresses the glial-derived neurotrophic factor (GDNF) that likely acts as a short-range attractive cue for the axons (Schuster et al., 2010). High local levels of GDNF tow these early axons along as the primI migrates all the way to the tail of the fish. Following neurons begin to extend their axons after primI has migrated some distance. These axons arrest elongation earlier and innervate more anterior neuromasts. Increasingly younger neurons first project their axons as the primI is further away. They arrest elongation even earlier to innervate the most anterior primary neuromasts (Pujol-Martí et al., 2010). The second wave of neurogenesis occurs coincidentally with the formation of the primII and primD primordial (Sarrazín et al., 2010). The axons of these new neurons follow these primordia and innervate their neuromasts. Some younger neurons also innervate primary neuromasts. At the end of the process, primary (older) neuromasts are innervated by first- and second-wave neurons, whereas secondary (younger) neuromasts are only innervated by neurons from the second wave (**Figure 2A**; Pujol-Martí et al., 2012).

### ORGANIZATION OF THE LATERALIS AFFERENT NEURONS

Pioneering neuroanatomical studies have shown that neurons that innervate anterior neuromasts project central axons to ventrolateral locations in the hindbrain, whereas neurons innervating more posterior neuromasts project dorsomedially (Alexandre and Ghysen, 1999). Therefore, it appears that the lateral line builds a continuous neural map by which the position of the lateralis afferents’ central axons along the dorsoventral projection column in the hindbrain reflects the spatial distribution of the neuromasts in the periphery (**Figure 2A**; Alexandre and Ghysen, 1999; Gompel et al., 2001; Luo and Flanagan, 2007). This type of neural-map organization receives the name of somatotopic. Of note, although some authors rightly point out that the lateralis neural map should be called “neurotopic,” we shall continue to (mis)-call it somatotopic because this definition appears in the bulk of the literature (Gompel et al., 2001). Live videomicroscopy combined with sparse fluorescent labeling of lateralis afferents showed early morphological and behavioral heterogeneities within the neuronal population, which correlate with their final central-projection patterns (Gompel et al., 2001). This result suggests that each neuron is pre-specified to occupy a particular position along the somatotopic axis. Importantly, cellular birth dating showed that neuronal morphology, behavior and projection pattern correlate with the time of neurogenesis (Liao, 2010; Pujol-Martí et al., 2010; Liao and Haehnel, 2012). During the first wave of neurogenesis, early differentiating lateralis afferents project dorsal central axons and innervate posterior neuromasts, whereas late-differentiating neurons project central axons more ventrally and innervate anterior neuromasts (**Figure 2**). These results suggest that neurogenic timing defines lateralis somatotopy (Pujol-Martí et al., 2010). Very recent observations have shown a more complex ordering of the lateralis central and peripheral axons (Pujol-Martí et al., 2012). Axons from second-wave neurons always occupy more ventral positions in the central-projection column than axons from first-wave neurons. To maintain somatotopy, second-wave neurons must project peripheral axons exclusively to anterior neuromasts. However, these neurons invariably innervate anterior as well as posterior neuromasts. Therefore, somatotopy is evident among neurons derived from the first wave of neurogenesis. When the whole population of lateralis afferents is examined simultaneously, the simple somatotopic ordering is lost (**Figure 2**).

### ARCHITECTURE OF THE LATERAL-LINE NEURAL MAP

The distribution and structure of the neuromasts enable the lateral line to decompose a complex hydrodynamic stimulus into its basic components: location, direction, and velocity. First, each neuromast responds to mechanical stimuli in its proximity by capturing sensory information from a discrete location along the animal’s body (**Figure 1**). Second, parallel and perpendicular neuromasts are sensitive to water movements along two perpendicular axes. Third, hair cells of each planar polarity class maximally detect water motions occurring along a single vector. Fourth, the collection of neuromasts along the animal body captures time-resolved stimuli, which represents water flow velocity. Once all components of the mechanical stimuli have been extracted by the receptors, they must be accurately conveyed to the central nervous system (Bleckmann, 2008). One possible mechanism to achieve this feat involves that signal location, direction, and polarity are segregated in the population of first-order lateralis afferents neurons, to be relayed through separate channels, and subsequently encoded by the spatial arrangement of neuronal projections in the brain.

### THE LATERAL-LINE NEURAL MAP IS HETEROGENEOUS

Very recent neuroanatomical work has re-explored the projection patterns of the lateralis afferent neurons in the zebrafish larva, putting emphasis on their connectivity (Faucherre et al., 2010; Liao, 2010; Haehnel et al., 2012; Liao and Haehnel, 2012; Pujol-Martí et al., 2012). These studies have revealed two classes of lateralis afferents in the posterior lateral line (Liao and Haehnel, 2012; Pujol-Martí et al., 2012). The first neuronal sub-class projects central axons to contact the lateral dendrite of the Mauthner cell, a *bona fide* output neuron of the lateral line (**Figure 2B**; Pujol-Martí et al., 2012). These neurons always project dorsally along the central-projection column. A second sub-class is characterized by central axons that do not contact the Mauthner cell, and that occupy more ventrolateral positions within the central-projection column. Recent data also demonstrated that birth-order correlates with lateralis map dimorphism (Liao and Haehnel, 2012; Pujol-Martí et al., 2012). Early born, dorsal projecting neurons that solely innervate primary neuromasts converge on the Mauthner cell, whereas later-born neurons innervating primary and secondary neuromasts do not converge on the Mauthner cell. Therefore, whereas every neuromast is somatotopically represented in the hindbrain, the lateral line also appears to directly input to the Mauthner cell in a putatively non-somatotopic fashion for it to broaden the receptive field of this reticulospinal command neuron (Pujol-Martí et al., 2012). Although such signal input to the Mauthner cell would decrease spatial discrimination, it may be essential to effectively evoke fast escape responses. Thus, the existence of two neuronal projection patterns indicates that the lateralis neural map is dimorphic, combining structural attributes of both the continuous and discrete maps (Luo and Flanagan, 2007; Pujol-Martí et al., 2012). Collectively, these studies provide solid evidence for a key twofold contribution of progressive neurogenesis to the patterning of lateral-line neural map (Pujol-Martí et al., 2010; Pujol-Martí et al., 2012). First, it arranges somatotopy by representing the spatial distribution of the mechanosensory stimuli that is likely to be essential for the complex neuronal computations used for navigation. Second, it delineates a dimorphic map architecture, which might represent independent channels of sensory-information transfer used for navigation and reflexive escape responses. Altogether, these data are helping formulate a simplifying principle that posits time as a key determinant of neural-map development.

### THE LATERAL-LINE NEURONAL POPULATION IS HETEROGENEOUS

Recent anatomical and genetic studies have shown further dimorphism among posterior lateralis afferents neurons in the zebrafish larva. A “large” sub-class of lateralis afferents has bigger somata and larger-diameter peripheral axons than the “small” sub-class (Liao and Haehnel, 2012; Pujol-Martí et al., 2012). Both neuronal sub-classes are myelinated (Lyons et al., 2005). Because the conduction velocity of myelinated axons in vertebrates increases linearly with their diameter, the large neuronal class is likely to conduct signals faster than the small (Goldman and Albus, 1968). Electrophysiological recordings have shown that the largest lateralis afferent neurons are less excitable and have a lower spontaneous firing rate (Liao and Haehnel, 2012). Although these recording were performed at the level of the neuronal cell body and, therefore, do not probe directly the actual excitability at the site of initiation of the action potentials, the collective evidence strongly suggests that the posterior lateral line of the zebrafish larva contains neurons that display heterogeneous anatomical and physiological properties. The large sub-class of neurons has “low excitability and high conduction velocity,” whereas the small sub-class has “high excitability and low conduction velocity.” What is truly interesting is that this anatomical-functional sub-division correlates with the neuronal classification that defines lateralis neural-map dimorphism. This is because large neurons project central axons dorsally and directly contact the Mauthner cell, whereas small, ventrolateral-projecting neurons do not contact the Mauthner (Haehnel et al., 2012; Pujol-Martí et al., 2012). This further divergence could explain how mechanical stimuli elicit either behavior governed by the lateral line. Rheotaxis, shoaling, and prey tracking would rely on a divergent sub-map in which each “high excitability/low conduction velocity” lateralis afferent synapses with up to 60 output targets in the hindbrain. Innate reflex escape responses, by contrast, would be based on a convergent sub-map in which “low excitability/high conduction velocity” neurons directly contact the lateral dendrite of the Mauthner cell to send strong depolarizing inputs with very short latencies. Thus, the activation of the large sub-class might suffice to trigger an escape reaction. However, because some small lateralis afferent also converge on the Mauthner cell, the escape response may be triggered by the coincident input on the Mauthner cell by small and large neurons. This neural-map architecture would safeguard animals from startling upon stimuli that would depolarize one neuronal sub-class but not the other, and is reminiscent of the escape strategy of crayfish, in which mechanosensory stimuli activate parallel neuronal pathways with different reaction times to trigger the startle reaction only when arriving coincidently to an output command neuron (Mellon and Christison-Lagay, 2008).

### NEURONAL ENCODING AND CENTRAL REPRESENTATION OF STIMULUS LOCATION

Anatomical studies have shown that each lateralis afferent neuron innervates a single neuromast in the zebrafish embryo and larva (Nagiel et al., 2008; Faucherre et al., 2009). During these early stages, multiple innervations are infrequent, but when they occur it is among adjacent neuromasts (Nagiel et al., 2008; ). For example, a single neuron can innervate all the terminal neuromasts. Therefore, the receptive field of an individual afferent neuron is generally defined by the neuromasts it innervates. Therefore, somatotopy in the afferent pathway likely forms a neuroanatomical code of the external hydrodynamic field. However, no physiological studies have so far been conducted in the central nervous system of the zebrafish, and studies in other fish species have failed to find space-selective neurons in the MON or the TS (Kunzel et al., 2011; Voges and Bleckmann, 2011; Mogdans and Bleckmann, 2012). These results, if confirmed by more exhaustive studies, would call into question the relevance of somatotopy for upper-level encoding of the dynamics of the hydromechanic scene (Kaas, 1997; Weinberg, 1997). One alternative mechanism is that stimulus location might be encoded by signal patterns in spatially non-segregated central neuronal population.

### NEURONAL ENCODING AND CENTRAL REPRESENTATION OF STIMULUS DIRECTION

Recent anatomical and physiological analyses in the zebrafish larva have shown that each neuromast is innervated by at least two lateralis afferents, each making synapses with hair cells of identical orientation, effectively dividing the neuromast epithelium in synaptic compartments of planar polarity (Nagiel et al., 2008; ). This holds true even in the case of neurons innervating multiple neuromasts (Nagiel et al., 2008; Faucherre et al., 2009). By doing so the input from each hair-cell polarity group may be conveyed by separate channels to the central nervous system. Physiological studies in other fish species have shown evidence for MON and TS neurons that are sensitive to the direction of water flow. This may occur if vector-sensitive central neurons existed and received input exclusively from a single hair-cells polarity group (Bleckmann, 2008). Alternatively, the direction of a water flow might be encoded in the brain exclusively by relying on somatotopy (see previous section). For instance, central neurons receiving inputs from different neuromasts might perform spatiotemporal cross-correlations to determine the direction of water flow (Chagnaud et al., 2008).

## OUTLOOK

The optical transparency of the zebrafish, coupled with its external and fast development, its diverse genetic toolkit, and the simplicity of the lateral-line mechanosensory system provide a powerful paradigm to study the development and homeostasis of sensorineural maps, and how they underlie the generation of appropriate behaviors to the environmental context (Friedrich et al., 2010). We now outline several interesting and central questions in neurobiology that could be answered using this model system.

Does the timing of neurogenesis contribute to the architecture of a neural map? If so, would time play a permissive or an instructive role? Recent investigations in the zebrafish started to reveal some of the mechanisms underlying the establishment of the lateral-line neural map during development (Pujol-Martí et al., 2012). Several findings strongly suggest that neurogenic timing is contributing to this process, supporting what it has already been found in other sensory systems (Jefferis et al., 2001; Pearson and Doe, 2003; Petrovic and Hummel, 2008; Tripodi et al., 2011). One emergent possibility is that neurons acquire different properties on the basis of their birth- or differentiation-dates. In the case of the lateral line, each afferent neuron could have an intrinsic date-of-birth-given identity that determines its final projection patterns. For instance, lateralis neurons born at different times could express different combinations of proteins (molecular codes) that might account for connectivity specificity in the context of a Sperry-type chemoaffinity mechanism (Sperry, 1963). This would be analogous to molecular heterogeneities within the retina and the tectum that govern retinotopic map formation (Lemke and Reber, 2005). Alternatively, differential expression of guidance receptors and ligands might occur in neurons born at different times, which could account for axonal segregation before neurons reach their peripheral and central targets (Imai et al., 2009).

Neuronal diversity may indicate that the projection pattern is an intrinsic property of the neuron. However, progressive neurogenesis might instruct map formation without necessarily diversifying neurons. Topographic mapping in the visual system of arthropods appears to occur in this way (Flaster and Macagno, 1984). In the case of the lateral line, progressive neurogenesis could be permissive and simply give rise to identical neurons that differ in their final projection patterns because they extend axons at different times to encounter environments that change as a consequence of the continuous growth of the brain. Thus, the final projection patterns of each neuron might exclusively reflect the interaction between the status of the surrounding tissue at axon growth. In such a case, the position of each neuron within the map would be circumstantial, rather than given by intrinsic properties of the neuron. Regardless, a temporal code might help match lateralis afferents with second-order neurons by first-born lateralis afferents reaching the target area first and associate with the earliest-born second-order neurons, whereas lateralis and second-order neurons that are born subsequently would synapse progressively, repeating the process (**Figure 2B**). To test this possibility directly, it will be essential to generate transgenic animals to identify and visualize second-order neurons in the hindbrain, and to examine their connectivity. Experimentally, it will be interesting to pause transiently the extension of the central axons. If neural map topology shows no differences after this manipulation, one could argue that factors other than timing of neurogenesis or axonogenesis play a role in neural map formation. Of course, it is also possible that the temporal factor plays no essential role in neural map formation in the lateral line.

Retinotopic map formation, for example, relies on a combinatorial action of molecular gradients, neural activity, and axonal competition (Triplett et al., 2011). Recent observations indicate that neither evoked activity nor inter-class axonal competition is a major force behind the ordering of the lateralis central axons along the hindbrain central-projection column (Faucherre et al., 2010; Pujol-Martí et al., 2012). However, the impact of these processes in connectivity or map refinement remains unknown. The assembly of a sensory neural map represents a remarkable developmental challenge. However, how neural maps are remodeled with the growth of the animal, are maintained during adulthood, or repaired after damage are equally important questions for which we currently lack an answer.

Does neural map dimorphism reflect the existence of functionally distinct neurons, which may subtract different aspects of a complex stimulus to convey them to separate groups of second-order neurons in the brain? If this were the case, it would be analogous to the central representation of sub-modalities observed in other sensory systems. For instance, the mammalian vestibular system, which is morphologically and physiologically analogous to the lateral line, also contains two afferent channels, characterized by complementary physiological properties, which are suited for driving distinct vestibular-related behaviors (Eatock and Songer, 2011). Are there molecular heterogeneities among the lateralis afferent neurons, which could play a role in neural map assembly? Recent work has revealed that some morphological and behavioral differences within the lateralis peripheral axons correlate with the expression levels of a transcription factor involved in neurogenesis called NeuroD (Sato and Takeda, 2013). The authors of this study suggest that different levels of NeuroD could switch-on different genes to create neuronal heterogeneities with an ultimate impact on their projection pattern. However, the observed different levels of NeuroD could simply reflect different stages of neuronal differentiation. Testing these hypotheses would require functional studies by genetic loss- and gain-of-function approaches in a spatiotemporally regulated manner (Asakawa et al., 2008; Abe et al., 2011; Faucherre and López-Schier, 2011; Lawson and Wolfe, 2011). Reverse genetic approaches using gain- and loss-of-function of candidate genes that are known to drive the development of other neural maps may answer this question. In addition, specific mutagenic and gene-trap screens will provide an unbiased entry point to this problem (Scott and Baier, 2009; Abe et al., 2011).

Fish should not startle by non-threatening stimuli. Also, behaviors such as navigation, rheotaxis, and schooling necessitate continuous input. It transpires that these contrasting behaviors would have different activating thresholds. Large neurons with low excitability and high conduction velocity that project from terminal neuromasts are well suited to produce the first and fastest lateral-line stimulus to the Mauthner cell, suggesting that terminal neuromasts have a disproportionate relevance in the escape behavior. Unlike neurons from other parts of the lateral line, each large neuron innervates up to three terminal neuromasts, which may increase their depolarization probability. Are terminal neuromasts enough to trigger the escape response in the zebrafish larva? If so, it may present clear survival advantages because terminal neuromasts would suffice to trigger an escape reaction by sending strong depolarizing inputs to the Mauthner cell with very short latencies. At least for the goldfish, afferent neurons with different excitability and conduction velocities have been found in the posterior lateral line. It would be interesting to explore these questions by combining laser-mediated neuronal ablation on transgenic animals, electrophysiological recordings to measure conduction velocities and patterns of neuronal excitability, and behavioral tests (Gahtan and Baier, 2004; Burgess and Granato, 2007; McLean and Fetcho, 2011).

One fascinating question is whether there are other sub-maps embedded into the lateral line. For instance, the second sub-class of lateralis afferents might assemble a second independent somatotopic map. Such a degree of complexity would not be unique to the lateral line. The visual cortex, for instance, contains a topographic representation of the retina and embedded in this map are multiple, superimposed maps of different stimulus attributes, such as eye dominance or motion direction preference (Swindale, 2001; Luo and Flanagan, 2007). A recent study on the organization of cutaneous low-threshold mechanoreceptors (LTMRs) in mice has shown that each hair-follicle type is innervated by a unique combination of LTMRs subtypes characterized by distinct physiological properties (Li et al., 2011). Although the neural map of the mammalian somatosensory system is somatotopic, the central projections of the distinct LTMRs subtypes terminate in different, yet partially overlapping laminae of the spinal cord dorsal horn, forming a discrete neural map in which neuronal subtypes are represented. The authors propose that such a neural map would allow for the integration of the individual mechanical properties of a tactile stimulus that takes place at a particular skin region. The structural similarity of their neural maps suggests that the mammalian LTMRs and the lateral-line sensory systems similarly integrate and process mechanical inputs.

What is the behavioral relevance of neural-map dimorphism? We have argued that the lateral line assembles a convergent/discrete sub-map for reaction speed, and a divergent/continuous sub-map for motor accuracy. This model could be tested using transgenic technology, cell biological and embryological manipulations. For example, laser nanosurgery on transgenic fish to ablate specific populations of neurons, and optogenetic actuators and sensors to excite or silence neurons, may be combined to probe their individual contribution of distinct subsets of lateralis neurons to a specific behavior. A detailed dissection of the connectivity patterns between the lateralis afferents and their central targets at the single-cell or whole-circuit levels will be essential to answer this question.

Another outstanding issue is the functional significance of the planar polarization of hair cells (López-Schier et al., 2004; Nagiel et al., 2008; Faucherre et al., 2009; Mogdans and Bleckmann, 2012). The existence of hair cells of two opposite polarities in the neuromast, and the somatotopic representation of the neuromasts in the brain, indicate that the lateral line may be able to localize mechanical signals along the animal’s body and discriminate the signals’ vectorial component (Faucherre et al., 2009; Faucherre et al., 2010; Mogdans and Bleckmann, 2012). Although to date there is no evidence supporting the idea that lateralis neurons collecting information from hair cells of opposite polarities establish connections with separate groups of second-order neurons in the brain, sensory information from signal location, direction and orientation may still be transmitted through different channels. If so, are these specific features of the sensory scene represented in maps or in clusters of similarly tuned high-order neurons? Where and how are they integrated? A combination of cell biology, genetics, electrophysiology, and optogenetics could help to unravel the functional role played by planar cell polarity in a sensory system.

## CONCLUSION

The zebrafish lateral line is emerging as a powerful model system to investigate how environmental cues are used to generate appropriate behavioral reactions to the sensory context. Future studies should combine physical and computational approaches to quantify the sensory landscape, genetic and optogenetic manipulations to dissect how the peripheral receptors extract and fractionate hydromechanical stimuli, and electrophysiological recordings to measure how the peripheral nervous system encodes and the central nervous system decodes stimuli. Such multidisciplinary approach will help to uncover and understand the mechanisms by which a sensory modality initiates and mediates contrasting behavioral programs.

## Conflict of Interest Statement

The authors declare that the research was conducted in the absence of any commercial or financial relationships that could be construed as a potential conflict of interest.
